# Microbiota Composition and the Integration of Exogenous and Endogenous Signals in Reactive Nasal Inflammation

**DOI:** 10.1155/2018/2724951

**Published:** 2018-06-03

**Authors:** Francesco Antonio Salzano, Luigi Marino, Giovanni Salzano, Riccardo Maria Botta, Giovanni Cascone, Umberto D'Agostino Fiorenza, Carmine Selleri, Vincenzo Casolaro

**Affiliations:** ^1^Department of Medicine, Surgery and Dentistry, “Scuola Medica Salernitana”, University of Salerno, 84081 Baronissi (Salerno), Italy; ^2^Department of Neurosciences, Reproductive and Odontostomatological Sciences, University of Naples “Federico II”, 80131 Naples, Italy

## Abstract

The prevalence of reactive nasal inflammatory conditions, for example, allergic rhinitis and chronic rhinosinusitis, is steadily increasing in parallel with significant environmental changes worldwide. Allergens and as yet undefined environmental agents may trigger these conditions via the involvement of host intrinsic factors, including the innate and adaptive immune system, the nasal epithelium, and the nasal nervous system. The critical role of the nasal microbiota in coordinating these components has emerged in recent studies documenting a significant association between microbial composition and the onset and progression of allergic or nonallergic inflammation. It is now clear that the local microbiota is a major player in the development of the mucosa-associated lymphoid tissue and in the regulation of such adaptive responses as IgA production and the function of effector and regulatory T cells. Microbial components also play a major role in the regulation of epithelial barrier functions, including mucus production and the control of paracellular transport across tight junctions. Bacterial components, including lipopolysaccharide, have also been shown to induce or amplify neuroinflammatory responses by engaging specific nociceptors. Finally, bacterial products may promote tissue remodeling processes, including nasal polyp formation, by interacting with formyl peptide receptors and inducing the expression of angiogenic factors and matrix-degrading enzymes.

## 1. Introduction

The nose, the uppermost portion of the respiratory tract, serves important physiologic functions, such as air filtration, warming, humidification, and olfaction. It consists of two cavities or *fossae* extending from the external nostrils (anterior nares) to the choanae and separated longitudinally by an osteocartilaginous septum. The lateral wall of each fossa provides insertion to three turbinates, or *conchae*, which divide the cavity in three passages, or *meatuses* [1]. These anatomical structures are essential to the air conditioning functions of the nose in that they expand the surface exposed to inhaled air. While the anterior nares and vestibule are lined with a skin-like stratified, keratinized epithelium, the nasal fossa proper is entirely coated with respiratory mucosa, consisting of a ciliated, highly vascularized, pseudostratified epithelium containing a sizeable number of mucus-producing goblet cells. The extensive vascularization of the nasal mucosa favors its air warming and humidifying functions, whereas the sticky seromucous secretions contribute to air filtering by effectively trapping inhaled particulate matter [2].

If the anatomy and physiology of the nasal cavities are complex, at least as complex are the pathophysiological processes that underlie the onset and progression of reactive nasal inflammatory conditions. These include a heterogeneous group of disorders, ranging from seasonal allergic rhinitis to nonallergic, persisting, refractory forms of chronic rhinosinusitis (CRS). About 400 million people worldwide are affected by allergic rhinitis, and another 200 million are thought to be affected by nonallergic forms of nasal inflammation including CRS [3, 4]. The overall prevalence of these conditions has been on a steady rise for almost 25 years concomitant with gross environmental changes in developed and developing countries [5]. While the inflammatory responses underlying allergic rhinitis are triggered by exposure to molecules with intrinsic allergenic properties, which promote type 2 T helper cell- (Th2-) biased, IgE-dependent immune responses, triggers of nonallergic rhinitis or CRS are nonspecific and largely unknown [4, 6]. Regardless, a number of common factors variably contribute to favoring and worsening the inflammatory response in these reactive nasal conditions [7–9]. These include the innate and adaptive immune system, the epithelial barrier function, a neuroinflammatory component (i.e., neurogenic inflammation), tissue remodeling processes, and the nasal microbiota.

In spite of the growing level of interest by the scientific community, still very little is known on the relationship between the nasal mucosal microenvironment, nasal allergic or nonallergic inflammation, and the nasal microbiota. Conversely, for a number of reasons, including the availability of suitable animal models, the central role of the microbiota in the coordination of the host homeostasis and specific disease processes is amply documented in several studies of gut immunopathology. In this review, we will touch on some of these studies in parallel with discussing more recent acquisitions in allergic rhinitis and related reactive nasal inflammatory conditions.

## 2. Towards the Definition of a “Healthy” Nasal Microbiota

The human microbiota, that is, the population of symbiotic microbes in the human body, has gained growing attention in the past few years, accounting for over 30,000 articles indexed in PubMed, over 25,000 of which published in the last five years [10, 11]. In recent years, studies of tissue-associated microbial communities have increasingly exploited the striking advances in next-generation sequencing and quantitative PCR of microbial genomes, or metagenomes [12]. Sequencing strategies vary greatly in different studies, the most common involving amplicon analysis of the 16S ribosomal RNA (rRNA), whereby bacterial operational taxonomic units (OTU) are mostly defined at the phyla or genera level depending on the sequence similarity threshold [13, 14]. However, coverage of larger, more complete sets of genes, as in whole-genome shotgun sequencing, is required to more accurately define microbial taxa down to the species and strain level and provide specific information on their physiological state, including the acquisition of accessory genes involved in virulence or antibiotic resistance [15]. Regardless of the breadth of coverage, current metagenomics tools have allowed to fully appreciate the extreme diversity of microbial communities and document the relationship between their imbalance, or *dysbiosis*, and seemingly unrelated disease processes, for example, obesity, autoimmunity, cancer, and mental disorders [12, 16–19]. On the other hand, they have allowed extending and overcoming most basic assumptions from earlier studies relying on semiquantitative cultures of bacterial colonies from fecal samples or other sources [12, 15]. It is now clear that distinct microbial communities exist on almost all epithelial surfaces of the human body [12, 13, 20] and that these consist of a highly diverse repertoire of bacterial, archaeal, viral, and fungal species [21–23]. In fact, recent estimates challenge the long-standing notion that a healthy human microbiota mostly consists of bacteria and that their numbers vastly exceed the total number of human cells [24]. Viruses, especially bacteriophages, are currently thought to outnumber the bacterial community by a ratio of at least 10 : 1 and contribute at least as substantially to the host homeostasis by acting on the bacterial phenotype and function or by directly interacting with the host mucosa [22, 23, 25].

Regardless of the association with clinical disease, the microbiota composition varies greatly in different individuals or different anatomical sites [12, 13]. As inferred in recent metagenomics studies of gut and oral samples from large populations sharing relatively common environments, interindividual variability largely reflects the environmental biodiversity rather than the host genetic background [26, 27]. On the other hand, the basis for intraindividual variability is still a matter of conjecture. The gastrointestinal microbiota has been investigated in most studies to date and is possibly the most abundant and diverse in the human body [13, 28]. Comparable OTU numbers, a measure of species richness, as well as disease-specific perturbations, for instance in patients with rheumatoid arthritis or cirrhosis, have been observed in distinct niches within the upper or lower gastrointestinal tract [29–31]. However, profound differences have been reported in the relative stabilities and recovery rates of commensal bacteria within the oral and gut mucosa following the administration of several classes of antibiotics [32]. Studies of the Human Microbiome Project cohort, in which 16S rRNA sequence clusters were examined at 18 different sites, have provided a possible explanation to these apparent discrepancies [13, 14]. In these studies, a *core* microbiota, defined as OTU shared across at least 95% of all samples for a given site, is identified as a stable, relatively ubiquitous, well-adapted microbial community, whereas noncore, *satellite* communities are identified at the subgenus level which are more variable across individuals, anatomical niches, time, and response to treatment [13, 14].

The nasal mucosa, given its affinities and contiguity to the lower respiratory tract and the sinus mucosa, the heterogeneity of its cellular components, its air conditioning and olfactory functions, and its permanent exposure to the external environment, represents a quite unique, attractive model for studies of host-microbe relationships in health and disease. The complex anatomy of the nasal cavities offers a highly diversified habitat to microbial species in a relatively narrow space [33, 34]. Most culture- or sequence-based studies of the nasal microbiota have until recently been limited to sampling the anterior nares and the vestibule, which exhibit similar overall histology to the external skin. Not surprisingly then, the distribution of certain phyla within this niche, namely, *Actinobacteria* and *Firmicutes*, as well as the overall richness in bacterial communities have been found to resemble those of the skin [13, 35]. A systematic study of 16S rRNA sequence clusters within two additional nasal sites besides the vestibule—the middle meatus and the sphenoethmoidal recess—identified in these mucosal sites a superimposable microbial colonization, consisting of about 50% *Actinobacteria*, 25% *Firmicutes*, and 20% *Proteobacteria* [33]. In contrast, the nasal vestibule was relatively enriched in *Firmicutes*, including *Staphylococcus aureus* [33]. A similar study identified almost 140 different taxa down to the species level by combining 16S rRNA sequencing and extensive culture of samples obtained during surgery from the anterior and posterior vestibule and the middle and inferior meatuses [34]. Core species identified at all sites by either approach included distinct *Staphylococcus* and *Corynebacterium* species, among others [34]. Similar core profiles were identified in a more recent study comparing the 16S rRNA amplicon sequence variants in samples from the anterior nasal cavity and the nasopharynx [36]. Aside from the relative abundance of species also detected in the lower respiratory tract, for example, *Streptococcus* and *Haemophilus* [37], nasopharynx samples from the majority of donors showed a more diverse “nasal” community, where *Corynebacterium*, *Staphylococcus*, and/or *Dolosigranulum* were the dominant core members [36].

## 3. Microbial Communities in Nasal Inflammation

Taken together, the findings outlined in the previous section are in line with the notion that, rather than being distributed in discrete niches, microbial communities throughout the nasal cavities and the upper respiratory tract may represent a continuum [36, 38]. This lends support to the idea, backing common clinical practice, that at least certain disease-specific associations can be recapitulated in swabs from a single site within the nasal cavities [39]. A number of studies have investigated the relationship between the nasal or nasopharynx microbiota and the frequency and severity of acute viral infections of the upper or lower respiratory tract (reviewed in [40]). A consistent association of 16S rRNA sequence profiles with disease severity was found in the anterior nares or nasopharyngeal swabs from over 800 infants hospitalized for bronchiolitis, whereby a *Haemophilus*- or *Moraxella*-dominated profile in either site appeared to be predictive of a higher or lower likelihood of intensive care use [39]. Along this line, a positive correlation between bacterial diversity, the relative abundance of *Haemophilus* and other species, and disease severity is documented in a metagenomics study of nasopharyngeal swabs from children hospitalized for influenza [41]. In contrast, a robust association was found between the frequency of symptomatic human rhinovirus (HRV) infections and a loss of microbial diversity in anterior nares swabs from an unselected cohort of 32 infants [42]. Evidence in support of a direct impact of viral pathogens on the nasal bacterial community is provided in a controlled study in which significant, long-lasting increases in *Staphylococcus* species relative abundance were seen in nasal swabs from healthy adult volunteers administered a live attenuated influenza virus vaccine [43]. Consistently, an up to 13-fold increase in *Staphylococcus* abundance was reported in the nasal lavage of volunteers subjected to experimental infection with HRV serotype 16 (HRV-16) [44].

Changes in microbiota composition subsequent to acute respiratory infections presumably reflect direct and diverse interactions of pathogenic viruses with the resident virome and bacteriome and/or the host immune system (reviewed in [45]). Studies in mouse models of influenza virus respiratory infection show that superinfection from *S. aureus* strains, including methicillin-resistant *S. aureus* (MRSA), is mediated by immune activation and the production of type I and III interferons (IFN) [43, 46]. Staphylococcal carriage within the nasal mucosa can be detected in about 30% of the general population and is a major risk factor for clinically significant, often severe infections of the lower respiratory tract, the skin, the bone, and other deep tissues [47–49]. An association between staphylococcal carriage, nasal dysbiosis, and nasal reactive inflammation was hypothesized by Salzano et al. in the early 1990s, who documented, using traditional culture methods and nasal challenges with bacterial antigens, the onset of more severe nasal symptoms concomitant with nasal colonization by *Chlamydia* and *Staphylococcus* species [50]. The relationship between nasal allergic inflammation and *S. aureus* carriage has been conclusively documented in a meta-analysis of ten studies conducted between 2000 and 2007: in nine out of ten studies, significantly higher numbers of adult or pediatric patients with allergic rhinitis were shown to test positive, at the local or systemic level, for *S. aureus* colonization [51]. A more recent 16S rRNA sequencing study documented increased microbial diversity in the middle nasal meatus of adult patients with seasonal allergic rhinitis and possible implications for airway inflammatory disease more in general [52]. Studies in patients with CRS, a more persisting form of nasal inflammation, presumably reflect the heterogeneity of this condition, as well as the protocols used for sample collection and processing, in that reduced or increased microbial diversity is detected from case to case; regardless, in most cases a relative enrichment is documented in staphylococcal species, especially *S. aureus* (reviewed in [53]).

Thus, studies of nasal inflammatory conditions of infectious or noninfectious etiology not necessarily fit the notion, inferred from studies of the gut or skin microbiota, that reduced microbial diversity, resulting from dietary changes, antibiotic overuse, and overall declining biodiversity, is most consistently associated with chronic disease, including allergic disease [54–56]. Conversely, clinical or subclinical infection with staphylococcal pathogenic strains emerges as a common denominator in the onset and progression of these conditions. Of note, superinfection by *S. aureus* strains in the context of a less diverse microbial community is a quite consistent finding in skin isolates from patients with atopic eczema (reviewed in [57]). Whether staphylococcal outgrowth in the atopic skin or nose is an initiating event that affects the relative abundance of symbiotic species or, rather, the result of changes in the microbial environment induced by other factors has yet to be determined. Such changes presumably reflect complex interactions between a genetically biased, imbalanced host response and more or less identifiable environmental signals. Substantial changes in the respiratory microbiota, staphylococcal outgrowth, and the development of a Th2-driven inflammatory response are consistently seen in human and animal models of infection with HRV, influenza, and other respiratory viruses [41–44, 58]. Indeed, these viruses are well-known for contributing to the onset and exacerbations of rhinitis, CRS, and asthma (reviewed in [59]).

While a body of evidence from studies of the gut microbiota may be extrapolated to such other districts, as the oral, vaginal, and respiratory mucosa, clearly more studies are required, above and beyond association studies, to understand how changes in the nasal microbiota affect the local homeostasis in health and disease. On the other hand, intestinal dysbiosis can precede and be conducive to the development of respiratory allergy [56, 60]. A possible cause-effect relationship between enrichment in a clostridial gut symbiont, *Ruminococcus gnavus*, and allergic rhinitis was convincingly demonstrated in a recent study combining prospective findings in fecal samples from an infant twin cohort and a suitable mouse model of airway inflammation [60]. Moreover, interventions aimed at rebalancing enteric communities in gastrointestinal disorders, as the oral administration of probiotics or prebiotics, have proved beneficial in several studies of apparently unrelated conditions of the respiratory tract, including cystic fibrosis, allergic asthma, and rhinitis [61–64]. Thus, regardless of site-specific differences in the core microbiota lining the respiratory and gastrointestinal mucosa, connections must exist between these mucosal sites and the factors that regulate their homeostasis, which will be discussed in the following sections.

## 4. The Microbiota in the Development and Regulation of the Immune System

It was shown as early as in 1970 that Peyer's patches, the spleen, and the lymph nodes of mice hosted in a sterile environment are underdeveloped and do not contain germinal centers, resulting in reduced serum immunoglobulin levels, and that normal immune system development and function were restored following oral administration of *Salmonella paratyphi* A [65]. Such germ-free, or *gnotobiotic*, mice, lacking a microbial antigenic stimulus and presenting an immature immune system, are still a widely used *in vivo* model to dissect host-microbe and microbe-microbe interactions at the gut mucosa [66]. An alternative mouse model allows dissecting the contribution of at least certain bacterial commensals to the host immune response via the sustained administration of distinct classes of antibiotics [67]. Knowledge acquired in these overall study models represents the experimental basis for the dominant current paradigms on how these interactions regulate the immune response and other processes.

The gut microbiota contributes to shaping both the innate and adaptive components of the immune systems. These include the gut-associated lymphoid tissue (GALT), effector T cells, regulatory T cells (Treg), IgA-producing B cells and plasma cells, innate lymphoid cells (ILC), and resident macrophages and dendritic cells in the lamina propria [68, 69]. The development and function of Peyer's patches is a case in point, as it is the macroscopic epiphenomenon of a complex molecular process. A number of studies have focused on the mediators involved in the interaction between the gut microbiota and the production of IgA from B cells in Peyer's patches. It has been shown that the coadministration of retinoic acid and the Toll-like receptor- (TLR-) 4 ligand, lipopolysaccharide (LPS), a toxic by-product of Gram-negative bacteria also referred to as *endotoxin*, stimulates follicular dendritic cells to secrete B-cell activating factor (BAFF), the chemokine, C-X-C motif ligand 13, and transforming growth factor- (TGF-) *β*, which collectively act onto Peyer's patch B cells to promote class switch recombination and the production of IgA [70].

**Figure 1:**
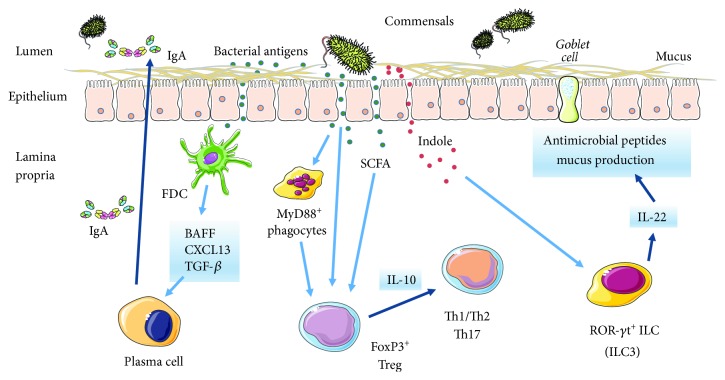
The regulation of immune responses by microbiota-associated factors. Follicular dendritic cells (FDC), myeloid differentiation primary response 88 (MyD88)^+^ phagocytes, and ROR-*γ*t^+^ ILC are stimulated by commensal bacteria to produce cytokines and other proinflammatory mediators. These responses involve, among other outcomes, the secretion of dimeric IgA, mucus, and antimicrobial peptides and are critically regulated by FoxP3^+^ Treg cells (see text).

It is well known that dimeric IgA are a fundamental effector arm of mucosal immunity and that IgA dimers produced from B cells activated in Peyer's patches play a significant role in the mucosal firewall and the prevention of infections both locally and at distant sites [71, 72]. Several lines of evidence, including studies in patients with selective IgA deficiency, suggest that proper IgA induction within the GALT also confers protection against allergic and autoimmune inflammatory conditions at distant sites [73, 74]. Accordingly, IgA-inducing, viable strains of *Bifidobacterium* and *Lactobacillus* have been shown to alleviate symptoms of pollen-induced rhinitis when administered orally at the onset of pollen season [62]. In addition, recent work has identified significant associations between the development of influenza-specific nasal IgA responses and the presence of such microbial species in the nasal mucosa, as certain *Lactobacillus* and *Bacteroides* strains [75]. While the GALT is considered the primary induction site for body-wide IgA production, antigen-specific, mucosal IgA responses can in fact be promoted in subjects administered an intranasal vaccine [76, 77]. The demonstration of comparable levels of IgA class switch by-products in Peyer's patches and the nasal mucosa provide factual evidence that the nasopharynx-associated lymphoid tissue may represent a primary IgA induction site (Figure 1) [78, 79]. While it was thought that primary antibody responses could only develop in secondary lymphoid organs, for example, lymph nodes and Peyer's patches, it is now clear that more or less organized ectopic or *tertiary* lymphoid tissue may form in the respiratory mucosa, where naïve B cells undergo class-switch recombination and the production of high-affinity antibodies (reviewed in [80]). This phenomenon is more accentuated in inflammatory conditions and on instances has been associated with the development of autoimmunity [80]. Of note, increased numbers of B cells and plasma cells, elevated levels of BAFF, and the local production of antinuclear autoantibodies of the IgG and IgA isotypes have been observed in the nasal polyps of patients with CRS [81, 82]. Naïve or IgA^+^ B cells in the nasal mucosa have also been shown to switch to IgE production in allergic rhinitis patients, which represents a conceptual basis in the appreciation of a subset of patients with local allergic rhinitis [8, 83].

Studies in gnotobiotic or antibiotic-treated mice have also provided evidence for a central role of the gut microbiota in the regulation of effector T cell responses. Selective depletion of gut-associated Gram-positive communities in neomycin-treated mice was shown to be sufficient to impair the airway innate and adaptive response to influenza virus infection, which could be rescued following rectal or nasal inoculation of a mix of TLR ligands [67]. Allergic airway inflammation is typically enhanced in germ-free animals as a result of skewed activation of Th2 clones, suggesting a major role for gut colonization in the development of a balanced type 1 T helper (Th1)/Th2 response [84]. Consistently, reduced Th1 responses can be observed in early infancy, especially in infants delivered by cesarean section, in whom an increased predisposition to develop allergic disease is associated with delayed gut colonization of *Bacteroides* species and a less diverse microbial community [85]. These and similar studies provide factual evidence in support of the *hygiene* hypothesis, according to which exposure to a declining environmental biodiversity is a major contributing factor in the increasing prevalence of allergic and other chronic inflammatory diseases as it adversely affects the human microbiota and its central functions in the development and regulation of the immune system [55].

Several studies have focused on the interactions of FoxP3^+^ Treg cells, a fundamental player in the immune regulatory network, with microbiota-delivered signals. A minimally diverse flora is required for activation and expansion of Treg cells and the production of interleukin- (IL-) 10, which involves the interaction of a host of bacterial components with distinct TLR or other pattern recognition receptors and depends on myeloid differentiation primary response 88-mediated signaling (Figure 1) [86, 87]. One such component is polysaccharide A from *Bacteroides fragilis*, which interacts with TLR-2 [88]. These effects, however, are apparently ligand-specific, as various TLR-2 ligands have been reported to either augment or decrease IL-10 production and Treg suppressive function [89]. Microbial metabolites, for example, butyrate and other short-chain fatty acids from *Bacteroides* and *Clostridium* species, can also direct the development and function of Treg cells and do so via the interaction with G protein-coupled receptors expressed by the intestinal epithelial cells and mucosal CD103^+^ dendritic cells [90, 91].

Induction and maintenance of a tolerogenic, Treg-dominated immune profile in the gut mucosa has been invoked to explain the beneficial anti-inflammatory properties of *Lactobacillus* and *Bifidobacterium* probiotic mixtures and of high-fiber diet [90, 92–94]. It is inferred, then, that an imbalanced diet would be associated with a dysbiotic microbial community and an augmented predisposition for inflammatory disease. Studies in germ-free mice have identified distinct bacterial species, for example, segmented filamentous bacteria, which can sustain autoimmune inflammation in models of arthritis and multiple sclerosis via the activation of IL-17-producing T helper (Th17) cells [95–97]. A more or less direct connection between diet, the microbiota composition, and allergic inflammation is postulated in several studies [64, 98]. Studies in germ-free and mutant mice have shown that the microbiota can regulate Th2-driven immunity through the induction of Th17 cells and of a subset of Treg cells expressing the Th17 hormone receptor, retinoid-related orphan receptor- (ROR-) *γ*t [99]. Symbiotic *Lactobacillus* strains and other bacterial species may directly activate these cells, as well as ROR-*γ*t^+^ type 3 ILC (ILC3), through the production of tryptophan-indole derivatives [100]. By engaging the aryl-hydrocarbon receptor, these metabolites induce in these cells the production of IL-22, a cytokine that promotes epithelial cell regeneration and the secretion of antimicrobial peptides and mucus, thus contributing to intestinal homeostasis (Figure 1) [100].

The beneficial effects of a properly balanced diet and/or the supplementation with oral probiotics in respiratory allergy support the notion that signals from the gut microbiota can shape local immunity at distant sites, including the upper and lower airways [64, 98]. In fact, evidence for a bidirectional crosstalk between the respiratory and gastrointestinal mucosa is provided in several studies [101, 102]. It has long been known that airway inflammatory changes of some sort are detectable in about 50% of patients with inflammatory bowel disease [101]. Conversely, gastrointestinal symptoms, including such disorders as eosinophilic esophagitis or gastroenteritis, are frequent comorbidities in children with asthma or allergic rhinitis (reviewed in [103]). This might reflect recirculation of inflammatory cells, for example, eosinophils, redirection of gut- or lung-homing antigen-specific cells, antigen cross-sensitization between the two sites, and/or concomitant changes in site-specific microbial communities (reviewed in [101]). However, changes in the nose and lung microbiota are found in patients with airway inflammation which hardly reflect the microbial environment in the gut [51, 52, 104, 105]. It has been shown that substantial changes in the lung microbiota, which also take place in the first few weeks of life, are sufficient to drastically reduce Th2-driven eosinophilic responses to aeroallergens by promoting the emergence of a Helios^−^ Treg subset via engagement of programmed death ligand 1 [106]. Nasal administration of *Lactobacillus* strains is sufficient to accelerate the recovery of functional humoral immune responses against respiratory pathogens in malnourished mice [107]. A reduced load of *S. aureus*, a pathogen commonly hosted in the inflamed nasal mucosa, and lower goblet cell counts are seen following intranasal administration of *S. epidermidis* [108]. Preclinical evaluations of similar probiotics preparations, which are shown to induce IL-10 expression in human PBMC, support the notion that they promote tolerance via the activation of distinct Treg cell subsets [109]. A decreased ratio of Treg to effector T cells can be appreciated in the inflamed nasal mucosa, as shown in adults with seasonal allergic rhinitis and, regardless of the allergic status, in those with CRS with nasal polyps (CRSwNP) or without nasal polyps (CRSsNP) [110, 111]. Consistently, significantly increased numbers of FoxP3^+^ Treg cells are seen in the nasal mucosa of adult patients with allergic rhinitis patients who successfully underwent grass pollen immunotherapy [112]. It can be envisioned then, as implicated in studies in mice administered nasal *Lactobacillus* probiotics carrying a specific allergen, that direct manipulation of the nasal commensal flora may significantly contribute to shaping the local immune response to promote antigen-specific tolerance via the induction of Treg cells [113].

## 5. Regulation of Epithelial Cell Functions by the Microbiota

Epithelial cells and their functions are well-established direct targets of microbiota-delivered signals. Epithelial cells lining the mucosa contribute to immune regulation via the production of cytokines and chemokines and by providing a dynamical barrier to corpuscular and molecular antigens present in the environment. The mucin layer that coats epithelial surfaces physically excludes commensal microbes [114]. Direct microbial contact with epithelial cell surfaces can occur in the absence of mucin layers or when specific microbes can penetrate mucin (Figure 1) [114]. Muc2 is the predominant mucin secreted by goblet cells in the intestine [115]. Two mucus layers are organized in the colonic mucosa, the innermost of which is dense and impenetrable to bacteria [114]. A more penetrable mucus layer and reduced barrier function have been detected in C57BL/6 mice being fed an autoclaved chow relative to wild or experimental mice on a standard diet [116]. This was associated with pronounced differences in the composition of the colonic microbiota at the class and genus level and could be reproduced in germ-free mice transferred with caecal contents from either diet group [116]. While the mechanisms of microbial regulation of mucus formation and stability have not been elucidated, it is likely that these reflect the differential abilities of bacterial strains to process and degrade carbohydrates of dietary or host origin, including Muc2-associated glycans [117].

The human nasal mucosa contains a substantial number of mucus-producing goblet cells. These are evenly distributed within the pseudostratified ciliated epithelium and are mostly concentrated in the maxillary sinus, where they represent about 40–70% of the surface cells [118]. Muc5B and Muc5AC are the prevailing mucins produced by the human respiratory epithelium and are produced at similar levels in the upper and lower airways [119]. Expression of both mucin genes has been found upregulated in the nasal and sinus mucosa of patients with CRSwNP or CRSsNP [120]. However, the pattern of mucin glycosylation appears to be altered in CRS and especially so in patients in which the nasal bacterial community assembles a biofilm [121]. While the cause-effect relationship of biofilm development and a dysfunctional mucus barrier has yet to be elucidated, it is well known that biofilm development favors antibiotic resistance and is associated with persisting inflammatory changes in the nasal and sinus mucosa, worse sinus symptoms, and poor clinical improvement following polyp removal [122]. One possibility is that increased mucin production in the absence of sufficient mucociliary clearance might lead to the formation of thickened mucus patches providing a favorable milieu for bacterial outgrowth, as previously postulated for *Pseudomonas aeruginosa* biofilm development in cystic fibrosis lungs [123]. Notably, a sizeable proportion of patients with refractory forms of CRSwNP are carriers of mutated alleles of the cystic fibrosis transmembrane regulator gene [124]. Besides *P. aeruginosa*, *S. aureus* is the most common isolate in biofilms from CRSwNP patients with relapsing disease after functional endoscopic sinus surgery [125].

**Figure 2:**
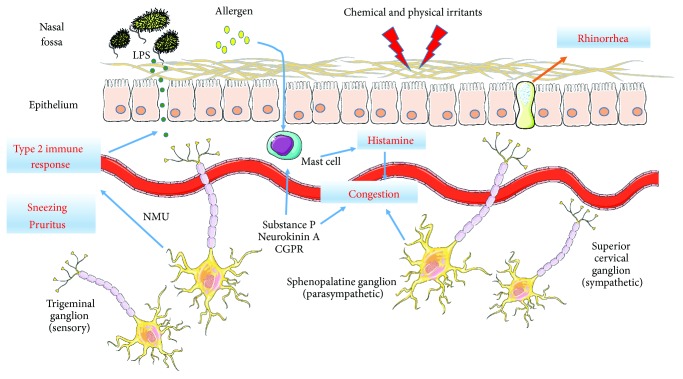
The relationship of allergen-induced, type 2 immune responses and the nasal nervous system. Trigeminal fibers are responsible for tactile sensitivity, including nociception, and release such neuropeptides as substance P, neurokinin A, CGPR, and possibly NMU (see text). These mediators induce vasodilation and directly activate cytotypes involved in the inflammatory response, for example, mast cells, eosinophils, lymphocytes, and macrophages. Parasympathetic postganglionic fibers release acetylcholine and induce vasodilation and mucus production, while norepinephrine from sympathetic fibers may induce vasoconstriction by interacting with *α*-adrenergic receptors, which typically prevails over vasodilation induced by concomitant ligation of *β*-receptors.

Factors produced by *S. aureus* can promote nasal inflammatory changes by directly acting on epithelial tight-junction (TJ) components to compromise mucosal barrier function [126, 127]. It has been documented that exposure of nasal epithelial cells grown at the air-liquid interface (ALI) to *S. aureus*-conditioned media determines a reduction of transepithelial electric resistance (TEER), a measure of barrier integrity, which is paralleled by the detection of a distinct separation between adjacent cells in the apical region, where TJ proteins are harbored [126]. These effects could be recapitulated upon exposure of these ALI cultures to staphylococcal V8 protease, which appeared to act on the assembly and expression of the TJ integral components, claudin-1 and *zonula occludens* protein- (ZO-) 1 [127]. As shown by Steelant et al. in two separate studies, the nasal epithelium from subjects allergic to dust mite or other inhalants expresses, both ex vivo and when cultured *in vitro*, reduced levels of occludin and ZO-1 and exhibits reduced TEER and increased permeability to fluorescein isothiocyanate-labelled 4 kDa dextran [128, 129]. Allergenic proteases, including dust mite major allergen, *Der* p 1, can at least in part account for these findings (Figure 2) [130]. TEER was further reduced in cells cultured in IL-4-supplemented media, suggesting that Th2-dependent immunity can affect the barrier function of the nasal mucosa [128]. In line with this notion, Saatian et al. demonstrated that the addition of IL-4 or IL-13 increases the permeability of airway epithelial cell monolayers *in vitro*, which is accompanied by the appearance of intercellular gaps and the accumulation of claudin-4 in cytoplasmic vesicles [131]. Besides Th2 cytokines, histamine, tumor necrosis factor-*α*, and the Th1 signature cytokine, IFN-*γ*, can also promote similar changes, including a decreased TEER and reduced claudin-1 and claudin-4 expression [129, 132]. Indeed, dysfunctional TJ and barrier function are not an exclusive feature of allergic inflammation but of chronic inflammatory processes in general [132].

The mechanisms of epithelial barrier disruption by pathogenic bacteria are not completely understood. It has long been known that certain bacterial toxins, for example, *C. perfringens* enterotoxin and *Vibrio cholerae zonula occludens* toxin (ZOT), may affect TJ integrity either by targeting its specific components or by binding to receptors shared with host-expressed homeostatic factors [133, 134]. One such factor, zonulin, shares with ZOT the ability to reversibly disassemble TJ complexes in the gut epithelium by interacting with protease-activated receptor-2 [135]. Zonulin may be induced upon exposure of small intestine monolayers to pathogenic enterobacteria or molecular patterns in certain nutrients, for example, gluten [136, 137]. Excess zonulin production, as a result of gluten intolerance or intestinal dysbiosis, and the ensuing loss of gut barrier integrity have been demonstrated in such chronic inflammatory conditions, as celiac disease and type I diabetes [135]. Less clear is zonulin involvement in the regulation of airway barrier function by the associated microbiota or aeroallergens. A molecule related to zonulin, presumably a serine protease, has been found to mediate albumin leak and complement activation in a mouse model of acute lunge injury [138]. An 8-mer peptide recapitulating zonulin and ZOT's effects on TJ complexes has the ability to increase the permeability of the nasal mucosa and help deliver antigen to the submucosa, thereby acting as an effective adjuvant for mucosal vaccines [139]. Thus, it is feasible that zonulin, or a closely related molecule, may play a critical role in the regulation of the nasal epithelial barrier and the nasal response to allergens, irritants, and toxins.

## 6. The Integration of Microbial and Neuroimmune Signals

Neurogenic inflammation owes to the dense sensory innervation of the nasal mucosa. The nasal mucosa is densely innervated with trigeminal fibers. Trigeminal nociceptors in the nasal mucosa consist of myelinated and unmyelinated fibers. The former, named A*δ*, transmit impulses faster than the slowly conducting, unmyelinated C fibers. C fibers mostly conduct nociceptive signals but also function as chemoreceptors in response to signals from transient receptor potential cation channel, subfamily A, member 1 (TRPA1), and transient receptor potential cation channel, subfamily V, member 1 (TRPV1), among others [140]. These are ionic channels that can be activated upon engagement by such specific ligands, as bradykinin to TRPA1 and lipid peroxidation or reactive oxygen species (ROS) to TRPV1. This implies that, besides sensory stimulation, also mediators of allergic inflammation can activate these receptors. The activation of TRPA1 and TRPV1 induces the inflow of cations and the antidromic depolarization of afferent fibers, also known as *axon reflex*. This in turn triggers the secretion, by the chemoreceptor itself, of such vasoactive mediators, as substance P, neurokinin A, neuropeptide Y, gastrin-releasing peptide (GRP), and calcitonin gene-related peptide (CGRP) (Figure 2) [141]. These neuropeptides contribute to vasodilation, glandular secretion, and lymphocyte and eosinophil effector function, resulting in such clinical symptoms, as congestion, rhinorrhea, sneezing, and headache, typical of nasal allergic inflammation (Figure 2) [142].

While nociceptive trigeminal fibers contribute to the development of allergic inflammation, they also directly affect nasal reactivity. In fact, the inhalation of irritating chemicals (gases, diesel particulate matter, etc.) induces more severe symptoms in allergic patients than in nonallergic controls, as shown in a 1998 study in which patients and controls were exposed to chlorine vapors [143]. Trigeminal nociceptors of allergic patients also have an increased activation threshold to tactile stimuli. We have detected a reduced tactile sensitivity of the nasal mucosa in elderly subjects following stimulation of the inferior turbinates with Semmes-Weinstein monofilaments [144]. Using a similar approach, we detected a reduction in the nasal tactile sensitivity of comparable grade in patients with allergic rhinitis compared to nonallergic controls [145]. Recent studies support a specific role for TRPA1 channels in the detection and response to microbial products. It has been shown that TRPA1 in vagal and somatic nociceptors can be activated by LPS, leading to the local release of such neuropeptides as CGRP, pain, neurogenic inflammation, and vasodilation (Figure 2) [146]. These effects of bacterial endotoxin are very fast, occurring within seconds of exposure and independently of TLR-4 ligation, and presumably involve specific structural features in lipid A, the LPS biologically active lipid moiety [146]. Similar results were obtained in a study in which distinct bacterial products, for example, N-formylated peptides, could mediate *S. aureus*-induced mechanical and thermal hyperalgesia in mice by directly activating nociceptors [147].

Taken together, these findings have led to the appreciation that bacterial products can induce a neuroinflammatory response independently of their interactions with the innate or adaptive immune system. The role of nociceptors in amplifying pathological immune responses to adaptive stimuli is in turn stressed in another study, in which ablation or pharmacological inhibition of Nav1.8-lineage neurons, which include the subpopulation of TRPA1^+^ nociceptors, decreased eosinophilia and macrophage accumulation in bronchoalveolar lavage fluids of mice subjected to allergen challenge [148]. One possible mechanism by which neurotransmitters can enhance the mucosal immune response to pathogens and allergens is suggested in recent studies showing that the neuropeptide neuromedin U (NMU), expressed in cholinergic neurons localized in the mouse gastrointestinal tract, can directly activate type 2 ILC (ILC2) through the interaction with the specific receptor, NMUR1 (Figure 2) [149]. NMU release occurred subsequent to direct sensing of parasite products and alarmins, and NMUR1-dependent induction of the cytokines IL-5, IL-9, and IL-13 in this model was found to promote accelerated parasite expulsion [149, 150]. Moreover, coadministration of NMU and the ILC2-activating epithelial alarmin, IL-25, strongly amplified airway inflammation in mice who underwent allergen challenge [151]. A similar crosstalk of neuroinflammatory signals in the nasal mucosa is suggested in studies showing that ligands of the GRP and neuromedin B receptors can interact with related or unrelated G protein-coupled receptors (GPCR), such as N-formyl peptide receptors (FPR), to promote mucus secretion, neutrophil recruitment, and the production of ROS [152, 153]. Thus, it can be envisioned that signals emanating from the microbial community may engage a complex interaction with immune and nervous system components within the nasal ecosystem as recently appreciated in the enteric mucosa [154].

## 7. Microbial Regulation of Tissue Remodeling in Nasal Inflammation

Morphologic alterations of the nasal mucosa are variably observed in patients with chronic inflammatory disorders of the nose and sinuses. These range from simple hypertrophy, mesenchymal transition, collagen deposition and fibrosis, polypoid degeneration, to polyps of various sizes and extension, resulting from different grades of tissue remodeling processes [9]. Tissue remodeling is defined by transient or permanent changes in tissue architecture, which involves breakdown of tissue structures, for example, basement membranes and interstitial stroma, as well as repair [155]. A pseudostratified, respiratory epithelium comprising ciliated and secretory cells and supported by basal cells lines the nasal and paranasal sinuses. In reactive nasal inflammation, its morphology is subverted and characterized by squamous metaplasia, ciliary destruction, increase of microfold cells, and mucous gland and goblet cell hyperplasia [9]. Although angiogenesis appears to be an important event in these processes, little is known about the mechanisms of vascular remodeling in the nasal mucosa. Numerous factors are dysregulated in the CRS mucosa which are involved in vessel remodeling, including TGF-*β*, platelet-derived growth factor, periostin, and vascular-endothelial growth factor (VEGF) [156–159]. Among these, VEGF could play a key role in polyp formation in CRS, thanks not only to its proedematous and angiogenic properties but also to its ability to promote nasal epithelial cell growth and resistance to apoptosis [156].

Several studies indicate that such innate immunity receptors, as FPR-1, FPR-2, and FPR-3, and matrix metalloproteases (MMP) may mediate the effects of microbial components on the tissue remodeling processes taking place in these conditions. The FPR are GPCR for the N-formylated peptides present in bacterial cell walls or in mitochondria and are expressed on the membrane of mononuclear and polymorphonuclear leukocytes. FPR ligation activates recruitment and activation of these cells via the engagement of phosphorylation cascades involving Akt, protein kinase C, and the Ras-mitogen-activated protein kinase pathway [160]. We hypothesized that engagement of FPR by bacterial ligands might be one possible mechanism linking nasal inflammation and dysbiosis to tissue remodeling processes leading to polyp formation. As shown by Prevete et al., the FPR agonists, f-Met-Leu-Phe and uPAR_84–95_, induce the migration of nasal epithelial cells *in vitro* and the production of VEGF and TGF-*β*, two factors involved in tissue remodeling [161]. A significant increase in VEGF expression, both at the mRNA and protein levels, and of MMP species involved in tissue remodeling, including MMP-2 and MMP-9, has been detected in fibroblasts from nasal polyps following *in vitro* infection with HRV-16 [162]. Consistently, in the nasal mucosa of patients with CRSwNP, we have detected a marked increase in the expression of MMP-2, MMP-7, and MMP-9, which was paralleled by reduced expression of tissue inhibitor of MMP- (TIMP-) 1 and TIMP-2 [163].

That tissue remodeling and polyp formation may be favored in a dysbiotic microenvironment is inferred in a number of studies showing increased *S. aureus* colonization in the nasal cavities of patients with polyposis [164]. Recent work has shown that *α*-toxin, one of the major *S. aureus* toxins, can substantially contribute to airway remodeling via a combined effect on the epithelial cell cytoskeleton and endothelial TJ integrity, leading, respectively, to altered morphology and edema [165, 166]. However, the main mechanism this far ascertained by which *S. aureus* may induce or favor nasal polyp formation is the production of such superantigens as staphylococcal enterotoxins (SAE) [7, 167]. SAE can bind to invariant domains of the T-cell receptor and of the major histocompatibility complex-II of antigen-presenting cells (APC) and induce the production of T cell and APC cytokines and other factors. More than 20 SAE have been described to date, the most studied being type A (SEA) and type B (SEB) staphylococcal enterotoxins [168]. It has been documented that exposure to SEB can induce the secretion of both Th1- and Th2-restricted cytokines, for example, IFN-*γ*, IL-4, and IL-13, from the healthy nasal mucosa. However, a polypoid nasal mucosa would release increased amounts of these cytokines when exposed to a comparable load of SEB, possibly reflecting priming by co-colonizing microbial species [167]. Among these, fungal species, for example, *Malassezia* and *Aspergillus*, are present at higher abundance in at least certain CRS phenotypes and may contribute to immune activation in the nasal mucosa via the interaction with lectin and antigen receptors (discussed in [169]). Consistently, it has been shown that the polypoid tissue contains increased amounts of the eosinophil-specific chemokine, C-C motif ligand-11, eosinophil cationic protein, IL-5, total IgE, and SEA- and SEB-specific IgE, relative to controls with CRSsNP [7].

**Figure 3:**
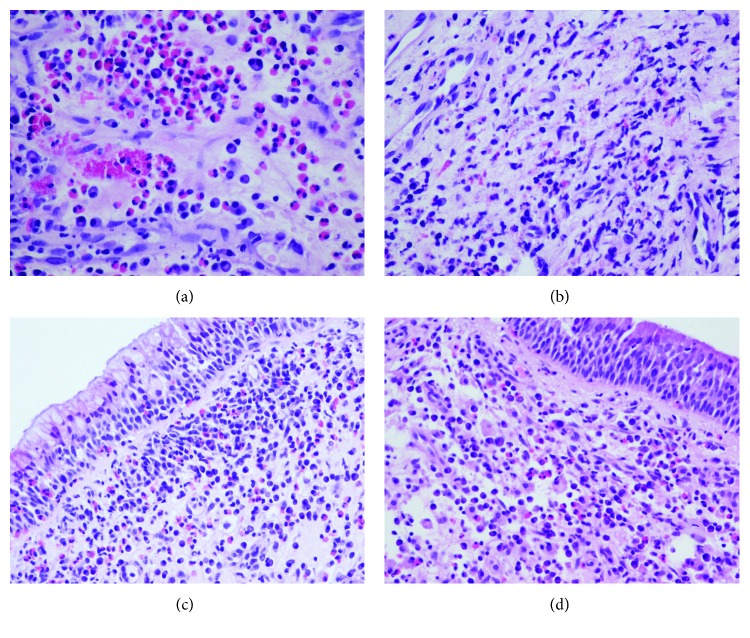
The effect of corticosteroid treatment on eosinophilic inflammation in CRSwNP. Hematoxylin and eosin stainings of polyp sections from two patients with CRSwNP before (a and c) and after (b and d) a short course of oral prednisone (0.4 mg/kg/day for 7 days) prior to polyp resection by functional endoscopic sinus surgery. Both patients did not have any allergies and were aspirin-intolerant. Polyp size and eosinophilic infiltration in the first patient (a and b), a 50-year-old female, were promptly reduced following prednisone administration, whereas polyp size and histology in the second patient (c and d), a 69-year-old male, remained substantially unchanged (F.A. Salzano and C. Stellato, unpublished observations).

Thus, while the mechanism of *S. aureus*-induced polyp formation has not been elucidated to date, it is current belief that this process is mediated by chronic Th2-dependent, eosinophilic inflammation in a *S. aureus*-colonized mucosa. In contrast, CRSsNP is mostly characterized by a predominantly neutrophilic infiltrate and a diverse Th1, Th2, and Th17 cytokine profile [170]. Remodeling in this condition is characterized by excessive collagen production and thickening of the extracellular matrix [157]. This process is mediated by TGF-*β*, which is distinctly upregulated in the CRSsNP mucosa relative to CRSwNP [157]. These findings present important therapeutic implications, in that, while Th2-driven eosinophilic inflammation and polyp formation are relatively well controlled with, and at least partly reversed by, inhaled corticosteroids, Th17-dependent inflammation and TGF-*β*-mediated remodeling are not [171–173]. However, numerous exceptions challenge these paradigms [170]. As shown in Figure 3, patients with apparently similar clinical and histological pictures may respond differently to medical and surgical treatment. The identification of discrete endotypes within either CRS phenotype is a growing need given the enormous potential of newly available targeted biotechnological therapies, for example, anti-IgE and anticytokine monoclonal antibodies (reviewed in [174]). An initial definition of up to 10 CRS inflammatory endotypes and their clinical correlates is provided in recent studies of expression clusters of cytokines and other biomarkers in the nasal cavities [175, 176]. One of these studies confirmed the strong association of SAE-specific IgE, a proxy for *S. aureus* carriage within the nasal mucosa, with nasal polyposis, measures of Th2-driven inflammation, and asthma comorbidity [175].

These results imply that distinct microbial signatures might be recognized across the expanding repertoire of CRS clinical and inflammatory endotypes. In a recent study comparing 16S rRNA sequences in paired swabs from the middle and inferior meatuses of adults with distinct nasal reactive inflammatory phenotypes, samples from patients with CRSsNP exhibited significantly lower overall microbial diversity relative to patients with CRSwNP, allergic rhinitis, and healthy controls [177]. Taxa enriched in CRSsNP relative to CRSwNP included *Streptococcus* and *Haemophilus* among others, whereas *Staphylococcus* and *Alloiococcus* were enriched in CRSwNP [177]. A separate 16S rRNA sequencing study of sinus brushings from a heterogeneous group of adults with CRS identified three main groups of patients based on bacterial community composition [178]. The largest group of patients, mostly with CRSwNP and a Th2-biased immune phenotype, exhibited a sinus microbiota reciprocally dominated by *Corynebacteriaceae* or *Staphylococcaceae* [178]. This confirmed that competing interactions may exist between these microbial families, whereby an increased colonization of a corynebacterial species, *C. pseudodiphtheriticum*, is associated with a reduced colonization of *S. aureus* and vice versa [33]. This led to hypothesizing that a *C. pseudodiphtheriticum*-colonized mucosa provides a less favorable microenvironment to *S. aureus* growth [33]. Besides competition for nutrients, *S. aureus* outgrowth in the nasal mucosa might be limited by the antimicrobial molecules produced by certain microbial species, as documented for species colonizing the intestinal mucosa [179]. One such species, the coagulase-negative staphylococcal strain *S. lugdunensis*, a common dweller in the human nose, produces a natural antibiotic, named lugdunin, which exerts a distinctive antimicrobial activity against *S. aureus* strains including MRSA [180]. Taken together, these findings emphasize the potential impact of interventions aimed at manipulating the nasal microbial community in the setup of a less favorable milieu for colonization from pathogenic species and ultimately the predisposition to develop nasal inflammation with different grades of such associated morphologic alterations, as hyperplasia, thickening, fibrosis, polypoid degeneration, or polyps of various sizes and extensions.

## 8. Concluding Remarks

The relationship between nasal dysbiosis and reactive, allergic or nonallergic, nasal inflammation involves a complex network of processes regulating mucosal permeability and TJ function, neurogenic signals, innate immunity cells and receptors, vascular and mucosal remodeling factors, effector T cells and related cytokines, and the production of specific IgE or IgA antibodies. The literature to date has not clarified the timing and reciprocity of these connections, and whether, for instance, intrinsic alterations in the mechanisms governing barrier function would typically precede or follow immune activation and inflammation. Moreover, the precise mechanisms that lead to distinct clinical phenotypes and endotypes and the inherent specific inflammatory processes are still largely unknown. Regardless, it can be concluded that the barrier function of the nasal mucosa, or *mucosal firewall*, represents the key element linking nasal dysbiosis to the cellular and molecular processes that lead to and sustain inflammation. An increased mucosal permeability may in turn favor bacterial translocation to the submucosa, where antigen presentation and recognition take place, as well as the interaction of bacterial components with innate immune and nociceptive receptors.

In light of these considerations, given the complex interactions between the microbial microenvironment, the nasal epithelium, the innate and adaptive immune system, and the nasal nervous system, it would be quite reductionist to classify nasal inflammatory processes based on the prevailing inflammatory cytotypes, for example, neutrophils, eosinophils, or mast cells, but should include at a minimum a definition of the immune phenotype or endotype to allow for a more targeted and effective line of intervention in the clinical management of patients with these conditions [174, 181]. In this light, even the resection of largely hyperplastic or extended, frankly polypoid lesions of the chronically inflamed nasal and sinus mucosa by minimally invasive, functional endoscopic surgery would not be seen as just the last resort whereby all other treatments have failed, but as the integral part of an organic strategy including conventional and biotechnological anti-inflammatory agents, antibiotics, and probiotics [182]. The possible effectiveness and appropriateness of probiotics in the management of at least certain CRS endotypes, and of reactive nasal inflammatory disorders in general, cannot be stressed enough. As confirmed in a study comparing the 16S rRNA sequence profiles before and after surgery of patients with refractory, relapsing forms of CRSwNP, conventional management of these patients with antibiotics and intranasal steroids is insufficient to prevent the rapid repopulation of the nasal mucosa with the baseline bacterial communities [183].

As discussed in this review, the rationale for probiotics administration in allergic rhinitis, CRS, and related nasal reactive disorders comes from studies documenting the antagonistic interactions of symbiotic and pathogenic species within the nasal mucosa or other niches [33, 108, 179, 180] and the ability of certain symbiotic species to regulate the fine balance between host immunity and tolerance [61, 75, 90–94, 100, 107, 113]. However, studies looking at the effects of oral probiotics on several clinical measures in pediatric patients with allergic rhinitis do not show consistent results [63, 184], and the findings from sparse reports in adults with CRS are discouraging [185]. On the other hand, the clinical use of nasal probiotics is supported in a number of preclinical studies and has shown promise in early-stage clinical studies in children with recurrent upper respiratory infections [107, 108, 186]. More studies are needed to fully understand the potential of this approach as a support treatment in nasal inflammatory conditions, for example, in allergic rhinitis patients undergoing specific immunotherapy and in surgical patients with resistant and relapsing forms of CRS.

In this review, we have focused on the possible mechanisms mediating disruption of the basic homeostatic functions of the human nasal mucosa concomitant to alterations in the local microbiota, which have been documented in nasal inflammatory conditions. Much of our knowledge comes from studies of gut bacterial communities, which provide a solid basis to understand the complex interactions between the host mucosa and the microbial milieu. Future studies will hopefully reveal how unique changes in the nasal microbiota, including viral and fungal components, result in distinct clinical phenotypes and how its manipulation may contribute to their current and prospective treatments.
